# Early diagnostic value of Serum GDF-15, RBP4 and SOCS3 in severe pneumonia with sepsis

**DOI:** 10.5937/jomb0-61152

**Published:** 2026-04-15

**Authors:** Mingying Luo, Ziyi Wang, Zhener Ke, Cong Fu

**Affiliations:** 1 The First Affiliated Hospital of Chongqing Medical and Pharmaceutical College, Department of General Practice, China; 2 Peking University People's Hospital, Department of Critical Care ICU, China; 3 Obstetrics & Gynaecology Hospital of Fudan University, Department of Critical Care Medicine, Shanghai Key Lab of Reproduction and Development, Shanghai Key Lab of Female Reproductive Endocrine-Related Diseases, China

**Keywords:** severe pneumonia, sepsis, growth differentiation factor 15, retinol binding protein 4, cytokine signal transduction inhibitor 3, early diagnosis, teška pneumonija, sepsa, faktor diferencijacije rasta 15, protein za vezivanje retinola 4, inhibitor signalne transdukcije citokina 3, rana dijagnoza

## Abstract

**Background:**

To explore the early diagnostic value of the serum levels of growth differentiation factor 15 (GDF-15), retinol binding protein 4 (RBP4), and cytokine signal transduction inhibitory factor 3 (SOCS3) in patients with sepsis caused by severe pneumonia (SP).

**Methods:**

A total of 110 SP patients were admitted to our hospital from December 2023 to February 2025. Based on whether they acquired sepsis, the patients were split into two groups: 52 patients with sepsis and 58 patients without. Additionally, 114 healthy individuals from the same age group who passed the health examination test at our hospital during the same period were selected as the control group. The levels of serum GDF-15, RBP4 and SOCS3 were detected via ELISA. Correlation analysis was conducted via the Pearson method. The factors influencing secondary sepsis in SP patients were examined using univariate and multivariate analysis. Serum GDF-15, RBP4, and SOCS3 levels were analysed for diagnostic effectiveness using a receiver operating characteristic (ROC) curve. The regional differences in the ROC curves of the serum samples were analysed via the paired comparison method.

**Results:**

RBP4 levels were substantially lower (P&lt; 0.05) while serum GDF-15 and SOCS3 levels were significantly higher in the study group. Compared with those in nonseptic patients, Serum GDF-15 and SOCS3 levels were considerably higher in SP patients in the sepsis group, whereas the level of RBP4 was significantly lower (P&lt; 0.05). The sepsis group's APACHE II score was substantially higher than the nonsepsis group's (P &lt; 0.05). The APACHE II score showed a favourable correlation (P&lt;0.05) with the levels of serum GDF-15 and SOCS3 in SP patients. The serum RBP4 level was negatively correlated with the APACHE II score (P&lt;0.05). Elevated levels of serum GDF-15 and SOCS3, along with increased APACHE II scores, are risk factors for sepsis in SP patients. In contrast, elevated serum RBP4 levels are a protective factor for sepsis in SP patients (P&lt;0.05). The area under the ROC curve (AUC) of the combined diagnosis of secondary sepsis in SP patients by serum GFF-15, RBP4 and SOCS3 levels was 0.946. The AUC of the combined diagnosis was superior to that of the individual diagnosis (Z= 1.970, 3.898, 3.188; P&lt;0.05).

**Conclusions:**

Serum GDF-15, RBP4, and SOCS3 levels in SP patients fluctuate in tandem with the severity of the patient's illness and subsequent sepsis. The combined diagnosis of these three factors has a certain value for secondary sepsis in SP patients.

## Abstract (sr)

**Uvod:**

Cilj je bio da se ispita rana dijagnostička vrednost serumskih nivoa faktora diferencijacije rasta 15 (GDF-15), proteina za vezivanje retinola 4 (RBP4) i inhibitora signalne transdukcije citokina 3 (SOCS3) kod pacijenata sa sepsom izazvanom teškom pneumonijom (TP).

**Metode:**

U studiju je uključeno ukupno 110 pacijenata sa TP hospitalizovanih u našoj bolnici od decembra 2023. do februara 2025. godine. Na osnovu prisustva sepse, pacijenti su podeljeni u dve grupe: 52 pacijenta sa sepsom i 58 pacijenata bez sepse. Kontrolnu grupu činilo je 114 zdravih ispitanika iz iste starosne grupe koji su tokom istog perioda prošli zdravstveni pregled u našoj bolnici. Nivoi GDF-15, RBP4 i SOCS3 u serumu određeni su ELISA metodom. Korelacija je analizirana Pearsonovim testom, dok su faktori rizika za sekundarnu sepsu kod pacijenata sa TP ispitani jednovarijantnom i multivarijantnom analizom. Dijagnostička efikasnost serumski nivoa GDF-15, RBP4 i SOCS3 procenjena je ROC krivom, a regionalne razlike analizirane metodom uparenih poređenja.

**Rezultati:**

Nivoi RBP4 bili su značajno niži (P &lt; 0 ,0 5), dok su nivoi GDF-15 i SOCS3 u serumu bili značajno viši kod pacijenata u grupi koja je posmatrana. U poređenju sa pacijentima bez sepse, pacijenti sa TP i sepsom imali su značajno više nivoe GDF-15 i SOCS3, dok je nivo RBP4 bio znatno niži (P &lt; 0,05). APACHE II skor grupe sa sepsom bio je značajno viši nego kod pacijenata bez sepse (P &lt; 0 ,0 5). APACHE II skor pozitivno je korelisao sa nivoima serumskog GDF-15 i SOCS3 (P &lt; 0 ,0 5), dok je nivo serumskog RBP4 bio negativno povezan sa skorom (P &lt; 0,05). Povišeni nivoi GDF-15 i SOCS3, zajedno sa povećanim APACHE II skorom, predstavljaju faktore rizika za sepsu kod pacijenata sa TP, dok povišen nivo RBP4 ima zaštitnu ulogu (P &lt; 0,05). Površina ispod ROC krive (AUC) za kombinovanu dijagnozu sekundarne sepse na osnovu nivoa GDF-15, RBP4 i SOCS3 bila je 0.946, što je značajno bolje u odnosu na pojedinačnu dijagnozu (Z = 1,970; 3,898; 3,188; P&lt;0,05).

**Zaključak:**

Serumski nivoi GDF-15, RBP4 i SOCS3 kod pacijenata sa TP odražavaju težinu bolesti i rizik od sekundarne sepse. Kombinovana dijagnoza ovih parametara ima značajnu vrednost za rano prepoznavanje sekundarne sepse.

## Introduction

Sepsis refers to severe and fatal organ failure that occurs in the body after infection. It is characterised by excessive activation of the coagulation system and upregulation of inflammatory responses, ultimately leading to disseminated intravascular coagulation (DIC) and vascular hyporesponsiveness [1][2]. In the early stage of disease, many inflammatory factors and mediators are released from lung tissue, which are amplified step by step in the body, triggering a severe inflammatory response, leading to severe pneumonia (SP), and further developing into sepsis [3][4]. Growth differentiation factor 15 (GDF-15) is a novel transcription factor with antiapoptotic, antiinflammatory and vascular endothelial protective functions that can regulate tissue repair and organ development and differentiation [5]. The rise in serum GDF15 levels is a prognostic indicator for sepsis, as it is directly linked to the severity and mortality of sepsis patients, according to studies [6]. The lipocalin family includes retinol-binding protein 4 (RBP4), which is mainly produced in adipose tissue and liver cells [7]. One study [8] confirmed that the serum RBP4 level in patients with sepsis is decreased. Combining the detection of RBP4 and procalcitonin can improve the diagnostic accuracy of sepsis. Numerous inflammatory and infectious disorders are linked to cytokine signal transduction inhibitor 3 (SOCS3), which negatively inhibits JAK/signal transducer and activator of transcription 3 (STAT3) [9]. Through the ADAR1-Mir-30a SOCS3 axis, studies have demonstrated that ADAR1 protects against sepsis by lowering organ damage and inflammation [10].

However, relatively few studies exist on the early diagnosis of SP-induced sepsis via GDF-15, RBP4 and SOCS3. Therefore, this study detected the levels of serum GDF-15, RBP4, and sOcS3 in SP patients and their diagnostic value in the early diagnosis of SP-induced sepsis. It should serve as a guide for the early detection and management of sepsis brought on by SP

## Materials and methods

### General case data

A total of 110 SP patients admitted to our hospital from December 2023 to February 2025 were selected as the research subjects. They were divided into 52 cases of sepsis and 58 cases of nonsepsis according to whether they progressed to sepsis within 24 hours of admission. The control group consisted of an additional 114 healthy individuals in the same age range who passed the health assessment test administered at our hospital during the same time period. This was done through a comparison and analysis of the fundamental data of the three patient groups, such as age and sex. The groups did not differ statistically significantly, and the comparison results were reasonable (P>0.05).

### Inclusion criteria

(1) The SP diagnosis met the relevant diagnostic criteria [11], and none of the patients had organ dysfunction before enrollment. (2) The diagnosis of sepsis conformed to the relevant diagnostic criteria [12]. (3) Patients over 18 years old, with a disease course of less than 24 hours and complete retention of serum samples and clinical data. (4) The patient and their family signed the relevant materials.

### Exclusion criteria

(1) Patients with malignant tumors, primary organ failure, blood system or immune system diseases; (2) Patients with primary severe extrapulmonary infection foci; (3) Those who have concurrent AIDS, are using immunosuppressants or have a weakened immune system for other reasons; (4) Pregnant and lactating female patients; (5) Patients with accompanying psychological disorders or cognitive impairment.

### Laboratory testing methods

3-5 mL of blood was collected intravenously on an empty stomach in the early morning using a serum separation gel procoagulant tube (BD Vacutainer SST II Advance, No. 367955, BD, USA). After blood collection, let it stand at room temperature for 30 minutes to allow it to coagulate fully. Serum was aliquoted into low-adsorption cryovials (Corning Cryovial, item No. 430488) and stored at -80°C for an extended period. Avoid more than one freeze-thaw cycle.

(1) R&D Systems Quantikine Human GDF-15 ELISA Kit, No. BMS2258 (R&D Systems, Bio-Techne, USA). Roche Elecsys GDF-15 (Electrochemiluminescence immunoassay, applicable to cobas e 601/801 platform; Roche Diagnostics).

(2) BioVendor Human RBP4 ELISA, No. MS2199 (BioVendor, Czech Republic).

(3) CUSABIO Human SOCS3 ELISA Kit, No. ab253890 (CUSABIO, Wuhan, China, the conventional detection range is mostly approximately 31.252,000 pg/mL).

### Detection of GDF-15, RBP4 and SOCS3 levels in serum

On the morning of the physical examination for healthy people and the second day following the patient's admission, 3 mL of venous blood samples were collected in an empty state. The levels of GDF-15, RBP4 and SOCS3 in the serum were determined via ELISA. The GDF-15 (R&D Systems, Bio-Techne, No. BMS2258), RBP4 (BioVendor, No. MS2199), and SOCS3 (CUSABIO, No. ab253890) used were purchased from Thermo Fisher Scientific Corporation of China and Abcam Corporation of the United States, respectively, and all the inspectors were trained professionals.

### Detection of sepsis-related marker levels in serum

The patient's serum levels of brain natriuretic peptide (BNP), procalcitonin (PCT), and C-reactive protein (CRP) were measured using ELISA. The PCT (No.: EHPCT), CRP (No.: KHA0031), and BNP (No.: EHNPPB) used were all purchased from Thermo Fisher Scientific China Co., Ltd.

### Statistical methods

The experimental results were statistically analysed via SPSS 25.0 software. Correlation analysis was conducted via the Pearson method. The factors influencing secondary sepsis in patients with SP were examined using univariate and multivariate analysis. Serum GDF-15, RBP4, and SOCS3 levels were analysed for diagnostic effectiveness using receiver operating characteristic (ROC) curves. The regional differences in the ROC curves of the serum samples were analysed via the paired comparison method.

## Results

### Serum GDF-15, RBP4 and SOCS3 levels in the study group and the control group

When comparing those in the control group with those in Table 1, the study group's serum GDF-15 and SOCS3 levels were noticeably higher, while the level of RBP4 was significantly lower (P<0.05).

**Table 1:** Levels of serum GDF-15, RBP4 and SOCS3 (pg/mL) in the study group and the control group.

Group	n	GDF-15 (pg/mL)	RBP4 (pg/mL)	SOCS3 (pg/mL)
Research Group	110	344.57±34.56	135.86±13.62	106.75±10.84
Control group	114	208.74±20.95	70.11±7.11	153.82±15.43
t value	-	35.713	45.517	26.332
P-value	-	≤0.001	≤0.001	<0.001

### Serum GDF-15, RBP4 and SOCS3 levels in patients in the sepsis group and the nonsepsis group

Serum RBP4 was considerably lower than that of the nonsepsis group (P<0.05), and the sepsis group had significantly higher levels of serum GDF-15 and SOCS3 than the nonsepsis group (Table 2).

**Table 2:** Levels of serum GDF-15, RBP4, and SOCS3 (pg/mL) in patients of the sepsis group and the nonsepsis group.

Group	n	GDF-15 (pg/mL)	RBP4 (pg/mL)	SOCS3 (pg/mL)
Sepsis group	52	371.85±38.69	63.64±7.21	169.21±21.95
Nonsepsis group	58	320.11±33.08	75.92±9.68	140.03±18.34
t value	-	7.559	7.475	7.592
P-value	-	<0.001	<0.001	<0.001

### Comparison of serum sepsis marker levels in patients with SP

As shown in Table 3, the SP patients' serum CRP and BNP levels did not differ statistically significantly (P>0.05).

**Table 3:** Comparison of serum sepsis marker levels in SP patients (pg/mL).

Group	n	PCT (pg/mL)	CRP (pg/mL)	BNP (pg/mL)
Sepsis group	52	76.85±7.71	36.53±3.68	202.78±20.36
Nonsepsis group	58	73.24±7.36	35.49±3.57	197.54±19.79
t value/X^2^ value	-	2.511	1.503	1.368
P-value	-	0.014	0.136	0.174

### Univariate analysis of secondary sepsis in SP patients

Age, sex, BMI, drinking, and smoking did not differ statistically significantly between the sepsis group and the coronary heart disease group, or WBC count between the sepsis group and the nonsepsis group (P>0.05). The APACHE II score of patients in the sepsis group was greater than that of patients in the nonsepsis group (P<0.05) (Table 4).

**Table 4:** Univariate analysis of secondary sepsis in SP patients (n(%), x̄ ± s).

Project	Sepsis group (n=52)	Nonsepsis group (n = 58)	X^2^ value/t value	P-value
Gender			0.014	0.905
Male	29 (55.77)	33 (56.90)		
Female	23 (44.23)	25 (43.10)		
Age (years)	63.3±8.4	64.5±8.5	0.801	0.425
BMI index (kg/m^2^)	22.89±2.32	23.61±2.51	1.557	0.123
Smoking			0.026	0.873
Yes	27(51.92)	31 (53.45)		
No	25(48.08)	27 (46.55)		
Drinking alcohol			2.401	0.121
Yes	21 (40.38)	32 (55.17)		
No	31 (59.62)	26 (44.83)		
Hypertension			0.553	0.457
Yes	27 (51.92)	26 (44.83)		
No	25 (48.08)	32 (55.17)		
Diabetes			0.050	0.824
Yes	28 (53.85)	30 (51.72)		
No	24 (46.15)	28 (48.28)		
Coronary heart disease			0.524	0.469
Yes	26 (50.00)	33 (56.90)		
No	26 (50.00)	25 (43.10)		
WBC content (x10^9^/L)	14.19±1.68	13.92±1.52	0.885	0.378
APACHE II Score (points)	19.87±2.17	16.41±1.84	8.965	≤0.001

### Correlation analysis of serum GDF-15, RBP4, and SOCS3 levels with the APACHE II score and sepsis marker levels

Serum GDF-15 and SOCS3 in SP patients were not related to the level of serum PCT (P>0.05). Serum GDF-15 and SOCS3 in SP patients were positively correlated with the APACHE II score, whereas the level of serum RBP4 was negatively correlated with the APACHE II score (P<0.05) (Table 5).

**Table 5:** Correlation between the levels of serum GDF-15, RBP4, and SOCS3 and the APACHE score and sepsis marker levels of patients.

Project	GDF-15		RBP4		SOCS3	
	r value	P-value	r value	P-value	r value	P-value
APACHE I Score	0.529	<0.001	-0.604	<0.001	0.777	<0.001
PCT	0.205	0.531	0.243	0.621	0.302	0.408

### Multivariate logistic analysis of the variables affecting SP patients' risk of developing secondary sepsis

The following independent variables were used: whether the patient developed secondary sepsis (yes = 1, no = 0) as the dependent variable; GDF-15, RBP4, SOCS3, and APACHE II scores; and PCT. These variables showed significant differences in the comparison results of general data, serological indicators, and serum sepsis-related markers. Patients with SP were analysed via logistic regression. The serum PCT level was not related to secondary sepsis in SP patients (P>0.05). Elevated levels of serum GDF-15 and SOCS3 and increased APACHE II scores were risk factors for secondary sepsis in SP patients, whereas elevated serum RBP4 levels were a protective factor against secondary sepsis in SP patients (P<0.05) (Table 6).

**Table 6:** Multivariate logistic analysis of the parameters that influence secondary sepsis in patients with SP

Influencing factors	value	SE value	Wald X^2^ value	OR value	95%CI	P-value
GDF-15	1.056	0.306	11.910	2.875	1.578~5.237	≤0.001
RBP4	-0.521	0.158	10.868	0.594	0.436~0.810	<0.001
SOCS3	1.076	0.306	12.357	2.932	1.610~5.341	≤0.001
APACHE I Score	1.137	0.312	13.285	3.118	1.692~5.747	<0.001
PCT	0.112	0.127	0.771	1.012	0.872~1.434	0.380

### Diagnostic value of serum GDF-15, RBP4 and SOCS3 levels for secondary sepsis in patients with SP

The areas under the ROC curves (AUCs) for serum GFF-15, RBP4, and SOCS3 in diagnosing secondary sepsis in SP patients, both alone and in combination (parallel method), were 0.890, 0.745, 0.795, and 0.946, respectively. The AUC of the combined diagnosis was superior to that of the individual diagnosis (Z=1.970, 3.898, 3.188; P<0.05) (Table 7).

**Table 7:** ROC analysis of serum GDF-15, RBP4 and SOCS3 levels for the diagnosis of secondary sepsis in SP patients.

Project	AUC	95%CI	Cutoff value<br>(pg/mL)	Sensitivity (%)	Specificity (%)	Ybuden Index
GDF-15	0.890	0.816~0.942	356.39	76.92	91.38	0.683
RBP4	0.745	0.653~0.824	72.180	82.69	60.34	0.430
SOCS3	0.795	0.708~0.866	155.29	61.54	86.21	0.478
United	0.946	0.886~0.980	-	94.23	84.48	0.787

## Discussion

Sepsis is a complex and heterogeneous syndrome with significant differences in clinical manifestations [13]. Pneumonia is one of the most common primary causes of sepsis in humans. Owing to the presence of endogenous endotoxins, SP accompanied by sepsis has the highest mortality rate in intensive care units worldwide [14]. During the course of sepsis, there is often a sharp increase in inflammatory factors, massive consumption of albumin, insufficient organ perfusion, severe immunosuppression, and even a gradual decline in immune function [15]. At present, in clinical practice, the treatment of sepsis caused by SP mainly involves drugs such as insulin, glucocorticoids, vasoactive drugs and anti-infective drugs [16]. However, in clinical practice, the efficacy of this therapy is not apparent. Moreover, after taking medication, patients often experience a series of relatively severe adverse reactions, which can have a certain impact on treatment [17][18]. This study explored the early diagnostic value of serum GFF-15, RBP4 and SOCS3 for patients with SP-induced sepsis.

It is usually only expressed in tissues like the prostate and placenta. However, its expression in tissues and blood dramatically increases under conditions of illness, such as inflammation [19]. Increased inflammation is linked to GDF-15, which also contributes to the synthesis of anti-inflammatory mediators. Relevant studies [20][21] have shown that GDF-15 has good clinical application value in sepsis. It can regulate the function of macrophages, inhibit the activation of the JAK1/STAT3 signalling pathway and the nuclear entry of NF-kBp65, and slow the progression of sepsis. The serum GDF-15 levels of SP patients in the sepsis group were much higher than those in the nonsepsis group, and they were positively connected with the patients' APACHE II ratings [22].

Most RBP4 is highly expressed in the liver, and most vitamin A is stored in the body in the form of retinol [23]. The increased concentration of intermediate atrial natriuretic peptide precursors of RBP4-related molecules in plasma may contribute to the development and incidence of sepsis [24]. The level of RBP4 in the serum of patients with sepsis is lower than that in the serum of nonseptic patients, and RBP4 can change insulin resistance in critically ill patients [25][26][27][28]. However, research on the early diagnosis of sepsis caused by RBP4 in SP patients is lacking. Serum RBP4 levels are protective against sepsis in SP patients and have a negative correlation with patients' Apache II scores [29]. RBP4 can reflect a patient's condition and provide a reference for the diagnosis of diseases [30][31][32].

Cytokine signal transduction inhibitor 3 (SOCS3) can negatively regulate the JAK-STAT signalling pathway. SOCS3 plays a role in immunological modulation, tumour progression, inflammatory response, and embryonic development [33][34][35][36]. Another study [37] indicated that SOCS3 was significantly highly expressed in the serum of patients with bacterial sepsis. The combined detection of PTX3, SOCS3, and sTREM-1 3 helps diagnose the occurrence of this disease. However, relatively few studies on the early diagnosis of SP-induced sepsis using SOCS3 are available.

ROC curve analysis in this study revealed that the AUC of the combined diagnosis of secondary sepsis in SP patients by serum GDF-15, RBP4 and SOCS3 levels was greater than that of the individual diagnosis, indicating that the combined diagnosis of secondary sepsis in SP patients has greater value and is highly important for the subsequent clinical treatment of patients. Timely detection of changes in serum GDF-15, RBP4, and SOCS3 levels helps guide clinical treatment.

## Conclusion

The expression of GDF-15 and SOCS3 in the serum of SP patients increases, while the expression of RBP4 decreases and correlates with the APACHE II score of patients. The combination of the three has specific value in predicting secondary sepsis in SP patients. However, this study still has certain limitations, such as a small sample size, insufficient variety of types, and insufficient depth of research. Further improvements will be made in the future to reduce errors.

## Dodatak

### Institutional review board statement

Our study does not contain data from any individuals or animals.

### Acknowledgments

The authors would like to express their gratitude to AJE and Yuesheng Press Language Editing Services for the expert linguistic services provided.

### Data availability statement

All relevant data are within the manuscript and its additional files.

### Conflict of interest statement

All the authors declare that they have no conflict of interest in this work.
